# A Brush With the Lake: Recurrent Lower Extremity Cellulitis in a 50-Year-Old Male

**DOI:** 10.7759/cureus.110524

**Published:** 2026-06-09

**Authors:** Lamisa Hassan, Caroline Aprigliano, Lee Pietrangelo

**Affiliations:** 1 College of Osteopathic Medicine, Touro College of Osteopathic Medicine, Great Falls, USA; 2 College of Medicine, Touro College of Osteopathic Medicine, Great Falls, USA; 3 Internal Medicine, Benefis Health System, Great Falls, USA

**Keywords:** cellulitis, freshwater exposure, gram-negative infection, myositis, soft tissue infection

## Abstract

Cellulitis is a leading cause of hospitalization in the United States. While β-hemolytic streptococci and *Staphylococcus aureus* predominate, patient comorbidities and environmental exposures may shift the spectrum of causative organisms. We report the case of a 50-year-old man with diabetes, prior left-leg deep vein thrombosis, chronic lymphedema, and recurrent cellulitis who presented with progressive lower-extremity erythema and pain following freshwater exposure. He failed multiple outpatient and inpatient empiric regimens and had non-diagnostic cultures. Magnetic resonance imaging demonstrated gastrocnemius myositis. Given characteristic dermatological morphology and exposure history, antipseudomonal therapy was initiated on hospital day four, resulting in rapid improvement. This case highlights the importance of integrating exposure history and attendant skin morphology to guide therapy in culture-indeterminate, refractory cellulitis and support antimicrobial stewardship.

## Introduction

Cellulitis is one of the most common skin and soft-tissue infections in the United States, accounting for over 14 million cases annually and representing a leading cause of infectious disease-related hospitalization [1]. Clinically, infections are categorized as purulent or non-purulent, a distinction that guides empiric antimicrobial therapy and reflects underlying microbiologic differences [2]. Although often discussed together when elaborating on non-purulent skin infections, a distinction can also be made between erysipelas, which involves superficial dermal lymphatics along with sharply demarcated borders, and cellulitis, which affects deeper dermal and subcutaneous tissues and typically lacks clear margins [1].

Despite its frequency, cellulitis remains a diagnostic and therapeutic challenge. Up to one-third of presumed cases are misdiagnosed, commonly representing venous stasis dermatitis, inflammatory dermatoses, or thrombotic disease, leading to unnecessary antibiotic exposure and avoidable hospitalizations [3]. While β-hemolytic streptococci and *Staphylococcus aureus* account for most infections, host factors and environmental exposures can substantially shift the causative spectrum [2]. Freshwater exposure in the setting of skin disruption introduces risk for Gram-negative organisms such as *Aeromonas* species and *Pseudomonas aeruginosa*, pathogens not reliably covered by standard empiric regimens [4,5]. Disruption of the cutaneous barrier allows direct inoculation of environmental microorganisms into deeper tissues, while prolonged water exposure may facilitate microbial colonization and impair normal wound healing. Seawater and brackish-water exposures, human and animal bites, pet and other animal exposures, plants and vegetation, contaminated loofahs, and intradermal foreign bodies, among others, all increase the risk of uncommon infectious agents. Moreover, cultures can frequently fail to identify causative organisms due to prior antibiotics, inadequate sampling, or biofilm-associated infection [6].

The clinical impact of cellulitis extends beyond acute infection. Patients with diabetes, chronic edema, lymphedema, venous disease, or prior deep vein thrombosis (DVT) face heightened risk for recurrence, complications, and treatment failure [7]. In this context, timely recognition of atypical presentations and exposure-associated pathogens is essential. This case illustrates how careful integration of exposure history, clinical morphology, and response to therapy can refine empiric antimicrobial selection in culture-indeterminate cellulitis and help prevent prolonged inpatient courses.

## Case presentation

A 50-year-old man with a history of ipsilateral recurrent streptococcal cellulitis, prior DVT of the left lower extremity, type 2 diabetes mellitus, chronic lymphedema, hypertension, and class II obesity (body mass index (BMI) = 35-39.99 kg/m², normal BMI = 18.5-24.9 kg/m²) presented with rapidly progressive redness, warmth, swelling, and tenderness of the left lower leg. Two weeks before presentation, the patient had sustained a superficial abrasion from a traumatic brush with an inboard boat motor casing to the left lower leg that initially drained serous fluid but improved with topical bacitracin and which had nearly closed. Approximately one week later, he noted a fluctuant area posterior to the healing abrasion that spontaneously ruptured, followed by rapid proximal spread of erythema toward the knee. He also reported increasing pain and swelling without frank purulence. The patient owned a marine business and reported that he had spent multiple hours working with his legs submerged in shallow lake water, resulting in repeated freshwater exposure while the wound remained open and during early re-epithelialization. He was subsequently evaluated at an outside hospital and treated empirically with oral trimethoprim-sulfamethoxazole and clindamycin without improvement, prompting emergency department presentation at our hospital.

Physical examination revealed diffuse erythema extending from the anterior distal left leg to just below the knee, superimposed on chronic lymphedema (Figure 1). Vital signs revealed tachycardia upon admission; the patient was otherwise hemodynamically stable. The affected leg was warm and tender with mild-to-moderate posterior calf tenderness. His skin exhibited a honey-crusted appearance along with continuous, almost weeping, yellow serous drainage and focal blue-green pigmentation. Xerosis with scaling was noted around the ankle and proximal calf. Hyperkeratotic calluses were present on the plantar surface of the foot. His entire examination revealed no fluctuance, crepitus, skin necrosis, pain out of proportion, or pain with passive stretch. Distal dorsalis pedis and posterior tibial pulses were intact with capillary refill at the toes in less than two seconds, and sensation was preserved.

**Figure 1:**
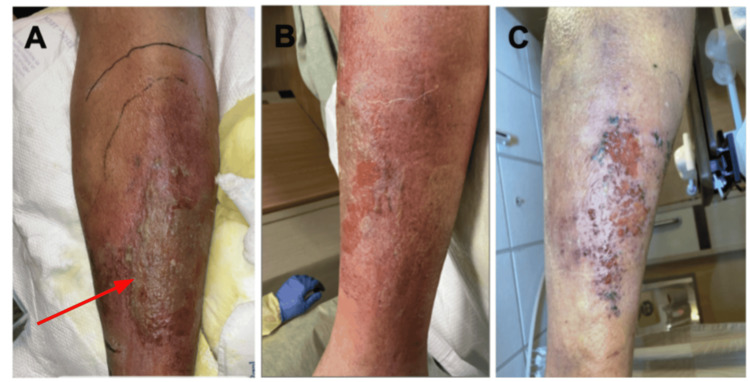
Clinical progression of left lower-extremity cellulitis. (A) Hospital day 0–1: Diffuse erythema and edema of the left lower extremity extending from the ankle to just below the knee with honey-crusting and focal blue-green pigmentation (arrow), a clinical feature that raised suspicion for Gram-negative infection. (B) Hospital day 3: Persistent diffuse erythema and swelling without significant clinical improvement, with progression of posterior calf tenderness and edema. (C) Hospital day 5: Marked clinical improvement with decreased erythema and edema and evolving brown scaling consistent with healing.

Laboratory findings demonstrated leukocytosis and elevated inflammatory markers, including C-reactive protein (CRP) and erythrocyte sedimentation rate (ESR), with gradual downtrending over the hospital course (Table 1). Hemoglobin A1c was 9.0% (normal range = 4.0-5.6%). The calculated LRINEC score was less than 3 [8]. Given the patient’s unilateral swelling and prior DVT history, venous duplex ultrasonography had been performed at the outside hospital and was negative for acute thrombosis. Necrotizing fasciitis and compartment syndrome were considered due to the severity and progression of pain but were deemed unlikely given the absence of systemic toxicity, neurovascular compromise, tense muscle compartments, crepitus, pain out of proportion, or pain with passive stretch. Osteomyelitis was also considered, but computed tomography and subsequent magnetic resonance imaging demonstrated no evidence of osseous involvement. The absence of sharply demarcated borders and the presence of apparent deeper tissue involvement favored cellulitis over erysipelas. Impetigo as a diagnosis was also considered due to the honey-crusted appearance of skin.

**Table 1:** Key laboratory findings. Note: Peak CK value was unavailable because serial CK measurements were not obtained. WBC = white blood cell count; CRP = C-reactive protein; ESR = erythrocyte sedimentation rate; CK = creatine kinase

Laboratory parameter	Initial	Peak	Final	Reference range
WBC (×10³/µL)	14.1	14.1	7.1	3.6–10.2
CRP (mg/L)	7.3	7.5	3.5	0.0–0.9
ESR (mm/hour)	100	100	39	0–20
CK (U/L)	64	N/A	35	55–170

The patient was admitted for severe cellulitis and started on intravenous vancomycin 1,750 mg, ceftriaxone 2 g, and clindamycin 900 mg (hospital days 0-1), with clindamycin added for anti-toxin effect given concerns over the severity of his skin and soft-tissue infection. Infectious disease consultation on hospital days 1-2 recommended continuation of ceftriaxone, substitution of doxycycline for vancomycin, and continuation of clindamycin; however, the patient showed no clinical improvement, and vancomycin was restarted on hospital day two. On hospital day three, the patient developed worsening severe posterior calf pain and an inability to bear weight. X-ray of the tibia and fibula showed no specific findings. Computed tomography of the tibia and fibula with intravenous contrast demonstrated diffuse subcutaneous edema and skin thickening without abscess or soft-tissue gas (Figure 2). Subsequent magnetic resonance imaging with and without contrast revealed myositis involving the medial and lateral gastrocnemius muscles without intramuscular abscess or deep fascial involvement (Figure 3).

**Figure 2:**
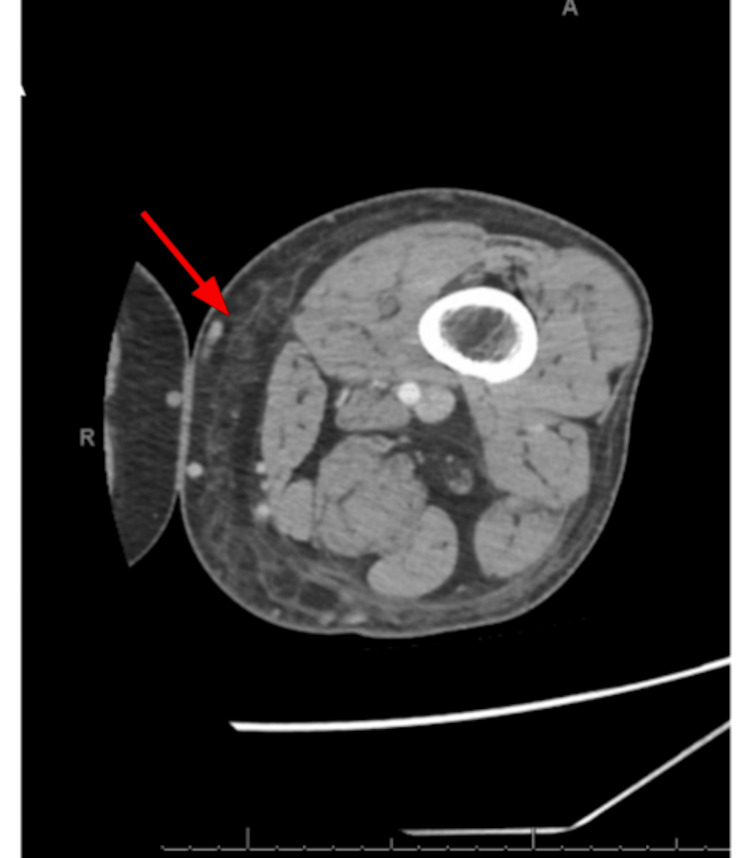
Axial contrast-enhanced computed tomography through the mid-calf of the lower extremity demonstrating diffuse soft-tissue edema (arrow) without evidence of gas formation or osseous involvement.

**Figure 3:**
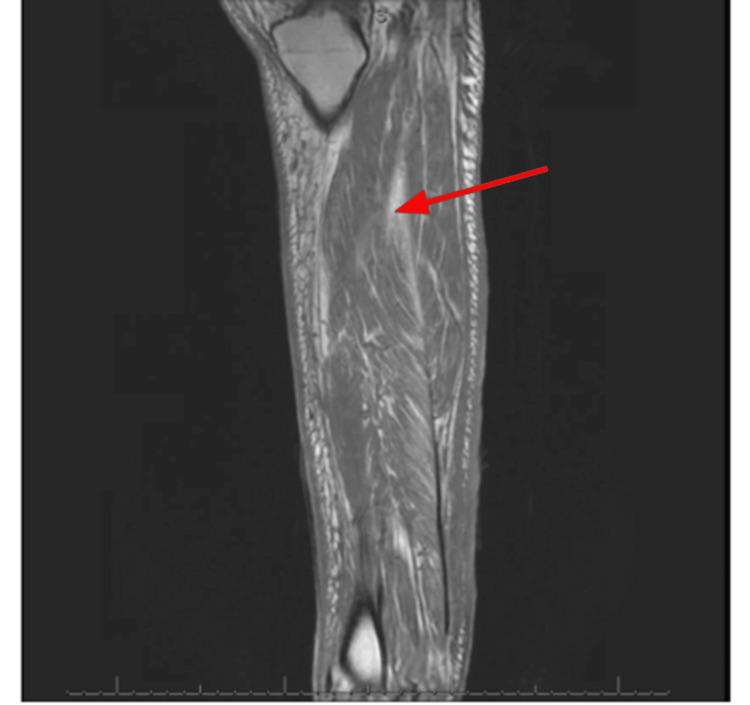
Magnetic resonance imaging of the lower extremity demonstrating intramuscular edema within the gastrocnemius muscle (arrow), consistent with inflammatory myositis without evidence of necrosis.

Given continued progression of infection and the relevant exposure history, antimicrobial therapy was broadened on hospital day four to include intravenous vancomycin 1,750 mg and piperacillin-tazobactam 4.5 g, with discontinuation of ceftriaxone and clindamycin. Once piperacillin-tazobactam was added, clinical improvement was observed within 24 hours, with continued improvement on hospital day five. Subsequently, the outside hospital wound cultures grew *Clostridium perfringens* after five days; however, this was interpreted as possible colonization or secondary contamination given lack of response to clindamycin and absence of gas, necrosis, or myonecrosis. Repeat aerobic, anaerobic, mycobacterial, and methicillin-resistant *Staphylococcus aureus* cultures remained negative. The patient was discharged on hospital day eight with oral levofloxacin 750 mg, doxycycline 100 mg BID, and metronidazole 500 mg BID to complete a seven-day course, along with instructions for wound care, continued compression therapy, and follow-up for diabetes management.

## Discussion

This case describes a 50-year-old man with multiple comorbidities presenting with recurrent, progressively severe cellulitis refractory to several empiric antimicrobial regimens. Despite broad initial coverage, the causative organism was not confirmed on culture, necessitating reliance on clinical morphology, exposure history, and disease trajectory to guide management. Wound cultures have limited diagnostic yield in nonpurulent cellulitis and are generally not recommended routinely, although they may be useful in atypical infections, immunocompromised hosts, or exposure-associated cases [1,2]. The patient exhibited persistently elevated inflammatory markers and developed severe gastrocnemius pain with impaired weight bearing, prompting further evaluation and ultimately revealing the additional diagnosis of myositis. While *Clostridium perfringens* infection was still possible, considering the outside hospital initial cultures, a freshwater-associated Gram-negative infection with probable pseudomonal involvement was considered most likely, although microbiologic confirmation was not obtained. The focal blue-green pigmentation and persistent serous drainage, together with freshwater exposure, history of diabetes, and failure of standard Gram-positive therapy, raised suspicion for a Gram-negative pathogen. *Aeromonas* species were also strongly considered given the freshwater exposure and are among the most commonly reported freshwater-associated wound pathogens. However, the focal blue-green pigmentation, clinical response to antipseudomonal therapy, and the patient’s diabetic risk profile raised suspicion for pseudomonal involvement, although definitive microbiologic confirmation was not obtained. Although *Clostridium perfringens* was isolated from the outside hospital wound culture, the absence of gas formation, necrosis, rapidly progressive toxicity, or response to clindamycin argued against clinically significant clostridial infection. The isolate was therefore interpreted as colonization or contamination.

Epidemiologic studies in diabetic populations identify Gram-negative organisms, including *Pseudomonas* species, as common isolates in diabetic foot infections, highlighting their relevance in metabolically compromised hosts [9]. This case illustrates exposure-driven cellulitis with imaging-confirmed myositis and stepwise escalation of antibiotics guided primarily by clinical features.

Several potential portals of microbial entry were present in this case, including chronic callus formation of the feet, minor skin trauma, and underlying lymphedema. Cellulitis occurs when microorganisms breach the cutaneous barrier through overt disruptions such as abrasions, ulcers, or fissures, as well as through less apparent portals, including interdigital maceration, chronic calluses, or lymphedematous skin with impaired barrier integrity [1,10]. Non-purulent cellulitis is classically associated with β-hemolytic streptococci, whereas purulent infections more frequently involve *Staphylococcus aureus* [2]. However, in patients with diabetes, obesity, or chronic edema, atypical and Gram-negative organisms are increasingly reported. Diabetes contributes to immune dysfunction through impaired neutrophil chemotaxis, reduced phagocytosis, and microvascular compromise, facilitating colonization and invasion by organisms such as *Pseudomonas aeruginosa* [11]. Consistent with such a risk profile, this patient’s hemoglobin A1c on admission was 9.0%, and glycemic control was addressed with inpatient insulin therapy. Poor glycemic control likely contributed to impaired host defense and increased susceptibility to Gram-negative infection in this case.

Imaging was pivotal in delineating disease extent and guiding escalation of therapy. Although routine imaging is not recommended for uncomplicated cellulitis, severe pain, inability to bear weight, and lack of clinical response warrant further evaluation. In this patient, computed tomography excluded abscess and necrotizing infection, while magnetic resonance imaging demonstrated gastrocnemius myositis. Infectious myositis has been reported as a complication of severe soft-tissue infection, particularly in patients with diabetes or immunocompromise, where delayed recognition is associated with substantial morbidity [12]. Gram-negative organisms such as *Pseudomonas aeruginosa* have been reported to cause invasive soft-tissue infections and myositis, particularly in metabolically compromised hosts [2]. Reports of cellulitis associated with freshwater exposure progressing to imaging-confirmed myositis remain rare in the dermatologic literature. Identification of myositis in this case supports the use of broadened antimicrobial coverage and underscores the importance of considering deeper soft-tissue involvement when clinical progression is discordant with initial therapy.

Therapeutic implications highlight the importance of early reassessment. Our patient failed multiple empiric regimens targeting typical Gram-positive organisms before demonstrating improvement only after initiation of piperacillin-tazobactam on hospital day four. While initial empiric therapy is necessary, prolonged exposure to serial broad-spectrum antibiotics increases the risk of antimicrobial resistance, adverse effects, and extended hospitalization [6]. Cultures can often be negative or indeterminate in skin and soft-tissue infections due to prior antibiotic exposure, low bacterial burden, or biofilm-associated infection [1]. Negative or unexpected culture results should be interpreted with appropriate discrimination. Early integration into decision-making of patient exposure history, skin and other clinical morphology, and disease severity may shorten hospitalization and allow for timely antimicrobial escalation when needed.

Recurrent cellulitis is strongly associated with chronic edema, lymphedema, and prior venous thrombosis, with diabetes being a strong risk factor [1]. Studies have demonstrated that patients with lymphedema or prior DVT experience significantly higher recurrence rates due to impaired lymphatic clearance and persistent tissue edema, which facilitate microbial persistence and reinoculation [7]. Preventive strategies, therefore, play a central role in long-term management. Adjunctive approaches aimed at improving lymphatic drainage, including osteopathic manipulative medicine, have been proposed for patients with chronic edema, although direct evidence supporting their role in cellulitis prevention remains limited [13]. Compression therapy remains the primary preventive intervention for recurrent cellulitis [14]. Meticulous skin care is equally critical, as hyperkeratosis and calluses can also serve as portals of entry for pathogens.

## Conclusions

This case underscores the importance of reconsidering microbiology and disease depth in patients with refractory cellulitis rather than repeatedly cycling through standard empiric regimens. Cultures may be unrevealing, and overreliance on negative results can delay appropriate therapy. In this patient, progression despite initial therapy and subsequent imaging-confirmed myositis suggested deeper soft-tissue involvement and prompted escalation of antimicrobial coverage, which was followed by rapid clinical improvement. Clinicians should consider freshwater-associated Gram-negative organisms, including *Aeromonas* species and *Pseudomonas aeruginosa*, particularly in patients with diabetes or chronic edema who fail standard Gram-positive empiric therapy. Patient education regarding wound care, glycemic control, compression therapy, and avoidance of environmental exposures during active skin disruption is essential to prevent recurrence. Recognition of exposure-associated pathogens and deeper soft-tissue extension can shorten hospitalization, improve antimicrobial stewardship, and guide targeted therapy in culture-indeterminate cellulitis.

## References

[1] Raff AB, Kroshinsky D (2016). Cellulitis: a review. JAMA.

[2] Stevens DL, Bisno AL, Chambers HF (2014). Practice guidelines for the diagnosis and management of skin and soft tissue infections: 2014 update by the Infectious Diseases Society of America. Clin Infect Dis.

[3] Nightingale R, Yadav K, Hamill L, Glasziou P, Scott AM, Clark J, Keijzers G (2023). Misdiagnosis of uncomplicated cellulitis: a systematic review and meta-analysis. J Gen Intern Med.

[4] Rutteman B (2017). Aeromonas wound infection in a healthy boy. JMM Case Rep.

[5] Makhdoomi MA, Abdo EM, Ilyas SO, Sedik AM, Elsayed AA (2019). Cellulitis left lower leg secondary to Pseudomonas aeruginosa bacteremia: case of community-acquired infection. Int Surg J.

[6] Obaitan I, Dwyer R, Lipworth AD (2016). Failure of antibiotics in cellulitis trials: a systematic review and meta-analysis. Am J Emerg Med.

[7] Ong BS, Dotel R, Ngian VJ (2022). Recurrent cellulitis: who is at risk and how effective is antibiotic prophylaxis?. Int J Gen Med.

[8] Wong CH, Khin LW, Heng KS, Tan KC, Low CO (2004). The LRINEC (Laboratory Risk Indicator for Necrotizing Fasciitis) score: a tool for distinguishing necrotizing fasciitis from other soft tissue infections. Crit Care Med.

[9] Patel TS, Cottreau JM, Hirsch EB, Tam VH (2016). Impact of hyperglycemia on outcomes of patients with Pseudomonas aeruginosa bacteremia. Diagn Microbiol Infect Dis.

[10] Norazirah MN, Khor IS, Adawiyah J, Tamil AM, Azmawati MN (2020). The risk factors of lower limb cellulitis: a case-control study in a tertiary centre. Malays Fam Physician.

[11] Zacay G, Hershkowitz Sikron F, Heymann AD (2021). Glycemic control and risk of cellulitis. Diabetes Care.

[12] Radcliffe C, Gisriel S, Niu YS, Peaper D, Delgado S, Grant M (2021). Pyomyositis and infectious myositis: a comprehensive, single-center retrospective study. Open Forum Infect Dis.

[13] Walkowski S, Singh M, Puertas J, Pate M, Goodrum K, Benencia F (2014). Osteopathic manipulative therapy induces early plasma cytokine release and mobilization of a population of blood dendritic cells. PLoS One.

[14] Webb E, Neeman T, Bowden FJ, Gaida J, Mumford V, Bissett B (2020). Compression therapy to prevent recurrent cellulitis of the leg. N Engl J Med.

